# Can Nucleobase Pairs Offer a Possibility of a Direct 3D Self-assembly?

**DOI:** 10.1186/s11671-016-1347-3

**Published:** 2016-03-09

**Authors:** Andrey N. Glushenkov, Dmytro M. Hovorun

**Affiliations:** Department of Molecular and Quantum Biophysics, Institute of Molecular Biology and Genetics, NAS of Ukraine, Zabolotnogo Str., 150, Kyiv, 03680 Ukraine

**Keywords:** Nucleobase pairs, Non-canonical base pairs, Non-planar base pairs, Nanobiotechnology, Cytosine-thymine, DFT, AIM, NBO, Self-assembly, 3D self-assembly

## Abstract

**Background:**

The nucleobase pairs are characterized by their conformational diversity in the wild. Yet a modern nanobiotechnology utilizes their planar conformations only, developing what can be called a “planar approach”. It is well established that the most energetically favorable conformations of the complementary nucleobase pairs are planar and correspond to the classical Watson-Crick nucleobase pairs.

**Presentation of the Hypothesis:**

The point of interest lies in a study of a conformational capacity of the nucleobase pairs to expand the diversity of a spatial configuration and to produce the complex 3D objects from the non-planar conformations. If such a goal could be achieved, then that could definitely open the perspectives for a novel “stereo approach”.

**Testing the Hypothesis:**

For the first time, basing on the first principles, we reveal an ability of the heteroassociates of the m^1^Cyt · m^1^Thy to form up to ten observable molecular complexes under standard conditions. The first three of them have population of ~90 % at standard conditions and are highly non-planar. The most energetically favorable structure has a T-shape, while the next two have an L-shape. At the same time, we show the lack of any experimental data covering a self-assembly of the m^1^Cyt · m^1^Thy base pairs.

**Implications of the Hypothesis:**

We present a theoretical evidence of the fact that the conformational capacity of the nucleobase pairs is much richer from the perspective of their self-assembly than it is considered in the modern nanobiotechnology. The capability of a modified cytosine and a modified thymine to create significantly non-planar structures opens a way for the innovative “stereo approach” to construction of the nanobiotechnological devices. We believe that a modern nanobiotechnological basis can and should be extended with the new nucleic base pairs with innate ability for non-planar structures. We would like to especially emphasize a prognostic role of our algorithm in obtaining the new results.

## Background

The modern nanobiotechnology uses the planar building elements for construction of the sophisticated synthetic DNA and RNA [1, 2]. Such elements are complementary base pairs of the adenine-thymine and the guanine-cytosine. This kind of approach allows the creation of a complex spatial structure from the separate modules called “tiles” [3–5]. The resulting structure is a bulk because of the need to bypass an immanent planarity of the complementary nucleobase pairs. In general, this approach originates in the historically acquired planar X-ray structures [6, 7], may be called “planar”. As one can see, this planar approach was determinative for choosing the base pairs self-assembly types that have been observed experimentally [8].

At the same time, the RNA shows a bigger diversity in the types of interacting base pairs [9] and employs both the canonical and non-canonical base pairs, which results in a rich variety of the secondary and tertiary structures of the RNA. It is obvious that the RNA crystal is capable of creating the non-canonical interactions as well as the non-planar forms of the nucleobase pairs by means of restriction of the degrees of freedom. This fact allows us to raise a question of the conformational capacity of the electron structures of the different nucleobase pairs. Does the free self-assembly of the essentially planar nucleic bases result in strictly planar base pairs or does it allow significantly non-planar structures as well? A positive answer to the question may lead to transition from the “planar approach” to the “stereo approach” that rely on the primary structure as the main source of an arbitrary secondary structure. Consequentially, it would allow the creation of more compact nanobiotechnological devices.

In our study [10], we prove that the model heteroassociates of the m^1^Cyt · m^1^Thy are capable of forming up to ten observable molecular complexes. The first three of them have population of ~90 % at standard conditions and are highly non-planar. The most energetically favorable structure has a T-shape, and the next two are of L-shape. Unfortunately, we failed to find any available published experimental data covering the self-assembly of the cytosine-thymine base pairs.

## Presentation of the Hypothesis

As it was mentioned above, the modern nanobiotechnology constrains itself by the use of only planar nucleobase pairs. In our opinion, it results from a relative impossibility to produce stable non-planar nucleobase pairs, so creation of some complex 3D structure out the innate planar elements requires usage of the sophisticated techniques. But if one were able to create the stable non-planar elements, achievement of the complex 3D structures might be much easier.

Taking into account the great conformational variety of the nucleobase pairs in the wild [9], as well as a relative lack of the theoretical awareness of all the possible conformations, we suppose that the nucleobase pairs are capable of forming observable non-planar structures. Because of a quite good knowledge of the complementary base pairs, we believe that those structures could be found in the non-canonical non-complementary nucleobase pairs. Additionally, we needed a means to find all possible conformations of the given nucleobase pairs. We solved that problem in [11].

## Testing the Hypothesis

Our hypothesis would be successful if we could find some theoretical grounds for any observable non-planar nucleobase pair that have not been revealed by the previous base-pairing experiments. And this paper testifies that we managed to find such a pair. The more important fact is that it has not been found experimentally yet.

The main goal of this paper is to obtain all possible observable m^1^Cyt · m^1^Thy heteroassociates for answering the question in the title. To fulfill this task, we used our own algorithm [11] of the input structures creating. We would like to emphasize the importance of the algorithm, which basically proceeds from an assumption that the nucleobase pairs could be stabilized by at least two intermolecular H-bonds. Aside from this assumption, the algorithm has no other restrictions and uses a modern view of the chemical nature of the H-bonds [12]. As a result, we obtained numerous optimized and stable non-planar structures of both the canonical and non-canonical nucleobase pairs. Although most of them are highly energetic complexes in the free form, we can use them for creating conditions in a crystal to achieve the exact desirable non-planar structure through restriction of the molecular degrees of freedom. That is how the wild-type RNA non-planar structures are created.

All the calculations have been carried out with the Gaussian 09 suite of the programs [13]. The relaxed geometries and their corresponding harmonic vibrational frequencies of the base pairs have been obtained using the density functional theory (DFT) with the B3LYP hybrid functional [14] for Pople’s 6-311++G(d,p) basis set in a vacuum. We performed the single-point energy calculations at the correlated MP2 level of theory [15] with the 6-311++G(2df,pd) Pople’s [16–18] basis set for B3LYP/6-311++G(d,p) geometries to consider the electronic correlation effects as accurately as possible.

The Gibbs free energy *G* values for all the structures were obtained at a room temperature (*T* = 298.15 K) in the following way:

1 $$ G = {E}_{\mathrm{el}} + {E}_{\mathrm{corr}}, $$

where *E*_el_ is the electronic energy and *E*_corr_ is the thermal correction.

Bader’s quantum theory “atoms in molecules” (QTAIM) was applied to analyze the electron density [19]. The topology of the electron density was examined using the program package AIMAll [20] with all the default options. The wave functions were obtained at the B3LYP/6-311++G(d,p) level of the theory. The presence of a bond critical point (BCP) of (3,-1) type [19] and a bond path between hydrogen donor and acceptor, as well as the positive value of the Laplacian at this BCP (Δ*ρ* ≥ 0), was considered as three criteria for the H-bond formation [19, 21].

Energies of the classical intermolecular H-bonds in base pairs were evaluated by the empirical Iogansen’s formula [22]:

2 $$ {E}_{\mathrm{HB}}=0.33\cdot \sqrt{\Delta v-40}, $$

where Δ*ν* is the magnitude of the redshift (relative to the free molecule) of the stretching mode of the H-bonded groups involved in the H-bonding. The partial deuteration, namely the partial deuteration of the amino group, was applied to eliminate the effect of the vibrational resonances [23].

Energies of the non-canonical so-called weak intermolecular CH..O/N H-bonds were evaluated by the empirical Espinosa-Molins-Lecomte (EML) formula [24, 25] based on the electron density distribution at the (3,-1) BCPs of the H-bonds:

3 $$ {E}_{\mathrm{HB}} = 0.5\times V(r), $$

where *V*(*r*) is the value of a local potential energy at the (3,-1) BCPs.

Relative strengths of van der Waals contacts were estimated by means of Grunenberg’s compliance constant formalism [26–28], calculated by the Compliance 3.0.2 program.

The main advantage of this approach is invariance of the compliance constants. The physical meaning of the compliance constants is deduced from their definition as a partial second derivative of the potential energy due to an external force:

4 $$ {C}_{ij}=\frac{\partial^2E}{\partial {f}_i\partial {f}_j}, $$

In other words, the compliance constants measure the displacement of an internal coordinate, resulting from a unit force acting on it. As follows from this definition, a lower numerical value of compliance constant corresponds to a stronger bond.

To study the charge transfer property in the interacting orbitals of the non-canonical intermolecular CH..O/N contacts, we used a natural bond orbital (NBO) analysis [29], which interprets the electronic wave function in terms of a set of occupied Lewis and a set of unoccupied non-Lewis localized orbitals. A second-order Fock matrix analysis was carried out to evaluate interactions between donor (*i*) and acceptor (*j*) bonds. The result of such interaction is a migration of the electron density from the idealized Lewis structure into an empty non-Lewis orbital σ^*^. For each donor (*i*) and acceptor (*j*) bond, the stabilization energy is

5 $$ {E}^{(2)}=\varDelta {E}_{ij}={q}_{ij}\frac{F{\left(i,j\right)}^2}{\varepsilon_j-{\varepsilon}_i}, $$

where *q*_*i*_ is the donor orbital occupancy, *ε*_*j*_ and *ε*_*i*_ are the diagonal elements, and *F*(*i*,*j*) is the off diagonal element of the NBO Fock matrix.

The atomic numbering scheme for nucleobases is conventional [30].

## Implications of the Hypothesis

We obtained the set of 51 complexes of the heteroassociates of m^1^Cyt · m^1^Thy. This set contains both the common and the rare tautomeric forms.

Total occupancy of the obtained complexes **1** (30.50 %) + **2** (26.66 %) + **3** (22.90 %) + **4** (7.64 %) + **5** (3.80 %) + **6** (3.09 %) + **7** (1.93 %) + **8** (1.14 %) + **9** (0.72 %) + **10** (0.67 %) constitute 99.04 % at normal conditions and defines all the experimentally observable complexes (see Table 1). For the purpose of achieving the declared goal, we will focus on these ten complexes in the rest of this paper.

**Table 1:** Common physico-chemical properties of observable heteroassociates of the m^1^Cyt · m^1^Thy

Complex	Δ*G* ^0^, kcal/mol	*μ*, D	H-bond AH…B/vdW cont. A…B	*ρ*, a.u.	Δ*ρ*, a.u.	100*ε*	*d*_AB_, Å	*d*_HB_, Å	Δd_AH_, Å	∠AHB, deg	E_HB_, kcal/mol
1	0.00	5.22	N3…С4	0.007	0.024	37.6	3.197	–	–	–	1.19
			N4 H…O4	0.021	0.078	7.6	2.997	1.991	0.010	169.3	3.87
			O2…N1	0.005	0.018	68.2	3.329	–	–	–	1.05
			C1H…O2	0.007	0.024	14.0	3.410	2.597	0.000	130.6	1.33
2	0.08	5.58	C1H…N3	0.011	0.033	11.1	3.340	2.488	−0.002	134.1	1.81
			N4H…O2	0.021	0.080	7.4	2.983	1.987	0.010	166.3	3.79
			O2…C6	0.002	0.008	101.0	3.716	–	–	–	0.34
3	0.17	5.65	N3…N1	0.006	0.020	110.8	3.314	–	–	–	1.16
			C1H…O2	0.008	0.023	8.7	3.554	2.584	−0.002	147.8	1.40
			N4H…O2	0.021	0.080	7.0	2.985	1.986	0.010	166.1	3.78
			C1H…N3	0.007	0.025	41.9	3.276	2.762	−0.002	108.5	1.30
4	0.82	2.91	N3H…N3	0.029	0.080	6.5	2.991	1.965	0.024	170.1	6.52
			N4H…O4	0.031	0.107	3.6	2.877	1.855	0.016	174.4	5.28
			O2…O2	0.002	0.008	23.1	3.714	–	–	–	0.33
5	1.23	2.06	N3H…N4	0.039	0.095	6.4	2.875	1.835	0.032	173.1	7.67
			N3H…O2	0.028	0.101	4.2	2.902	1.879	0.017	172.7	5.25
6	1.36	4.96	N3H…N3	0.029	0.079	6.4	2.995	1.969	0.023	169.9	6.40
			N4H…O2	0.028	0.101	4.0	2.901	1.883	0.014	173.2	4.85
			O2…O4	0.002	0.007	39.0	3.773	–	–	–	0.29
7	1.64	1.26	N3H…N4	0.040	0.096	6.4	2.865	1.823	0.033	173.6	7.86
			N3H…O4	0.030	0.103	3.5	2.889	1.863	0.019	173.2	5.61
8	1.95	5.06	C1H…N3	0.014	0.043	4.6	3.411	2.332	−0.001	169.5	2.28
			N4H…O2	0.022	0.089	6.5	2.962	1.948	0.010	174.2	3.70
9	2.22	13.05	C6H…O2	0.013	0.043	2.1	3.335	2.284	0.001	162.6	2.43
			C5-1H1..N3	0.004	0.012	0.2	4.047	2.978	0.000	166.2	0.66
			C1H…O2	0.008	0.030	15.5	3.488	2.449	−0.004	159.4	1.59
10	2.27	13.57	C1H…O2	0.005	0.017	173.4	3.798	2.814	−0.004	150.3	0.93
			C1H…N3	0.006	0.017	5.9	3.791	2.745	−0.004	161.1	0.95
			C6H…O2	0.014	0.051	1.4	3.294	2.211	0.001	175.5	2.65

*ΔG* ^*0*^ relative Gibbs free energy (*T* = 298,15 K; *P* = 1 atm), *μ* dipole moment, *AH…B/A…B* atoms participating in H-bond and/or in van der Waals contacts, *ρ* electron density, *Δρ* Laplacian of electron density, *ε* ellipticity in bond critical points of (3,-1) type, *E*_HB_ H-bond energy, *d*_*AB*_, *d*_*HB*_,  *AHB* distances and angle between atoms of H-bond/van der Waals contact, *Δd*_*AH*_ elongation of AH group in H-bond

**Fig. 1:**
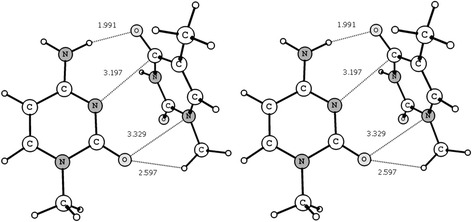
Stereo image of the most energetically favorable heteroassociate of m^1^Cyt · m^1^Thy. Its T-shaped and stabilized by means of two H-bonds and two van der Waals contacts (represented by *dotted line*; their corresponding bond lengths H…B/A…B are given in Å). Note that the 1-methyl group of m^1^Thy actively interacts with the m^1^Cyt

The most energetically favorable complex **1** has a T-shape structure and is stabilized by means of two intermolecular H-bonds and two attractive van der Waals contacts (see Fig. 1).

We have found out that the obtained heteroassociates of the m^1^Cyt · m^1^Thy are stabilized by means of the intermolecular H-bonds of NH..N/O, CH..N/O types and attractive van der Waals contacts of the N..N/O/C, O..O/C. Energies of the classical H-bonds lie in diapason of 3.70 ÷ 7.86 kcal/mol. Energies of the non-canonical H-bonds lie in diapason of 0.66 ÷ 2.65 kcal/mol.

**Fig. 2:**
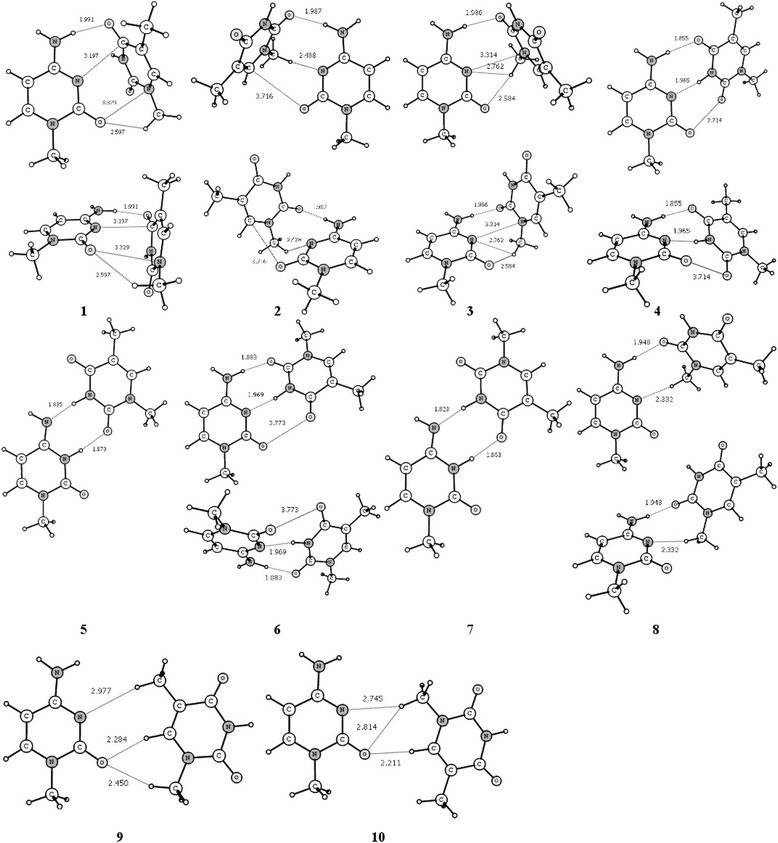
Geometrical structure of observable at normal conditions heteroassociates of the m^1^Cyt · m^1^Thy. The non-planar heteroassociates are represented in two projections while the planar ones are represented by one projection. *Numbers under heteroassociates* correspond to the row ‘complex’ of Table 1. H-bonds and van der Waals contacts (represented by *dotted lines* and their corresponding bond lengths H…B/A…B are given in Å)

The observable heteroassociates of the m^1^Cyt · m^1^Thy have several different shapes (see Fig. 2): the T-shape is represented exclusively by the most energetically favorable complex **1**, an L-shape (complexes **2**, **3**), a spiral shape (complexes **4**, **6**), the planar structures (complexes **5**, **7**, **9**, **10**), and a severely non-planar structure of the complex **8** which cannot be classified as one of above. As one can see, the most expected shapes are the L-shape (49.56 %), the T-shape (30.5 %), the spiral shape (10.73 %), the planar shape (7.3 %), and finally the complex **8** shape (1.14 %). This fact gives us the positive answer to the question in the title.

We believe that such unusual geometries are the result of a strong involvement of a methyl group in stabilization of the heteroassociates. As one can see from Table 1, the methyl groups (C1H, C5H) are capable of forming the strong enough CH..O/N H-bonds with energies in diapason of 0.66 ÷ 2.28 kcal/mol. At the same time, one can observe the strong stabilizing van der Waals contacts (see Table 2), which are present in five out of ten observable heteroassociates, and have energies in the diapason of 0.29 ÷ 1.19 kcal/mol. The especially strong van der Waals contacts are surveyed in the most energetically favorable complex **1** with the energies of 1.05 and 1.19 kcal/mol respectively.

**Table 2:** Intermolecular CH..O/N H-bonds and van der Waals contacts in the observable heteroassociates of the m^1^Cyt · m^1^Thy

Complex	H-bond/vdW contact AH..B/A..B	*E* ^(2)^, kcal/mol	*C*str, Å/mDyn
1	C1H…O2	0.36	39.549
2	C1H…N3	1.00	22.928
3	C1H…O2	0.76	37.566
	C1H…N3	0.03	33.407
8	C1H…N3	3.53	11.200
9	C6H…O2	3.27	16.085
	C5H..N3	0.55	66.521
	C1H…O2	1.00	27.809
10	C1H…O2	0.29	126.112
	C1H…N3	0.74	43.476
	C6H…O2	3.98	11.659

*E* ^*(2)*^ value of stabilization energy, *Cstr* linear Grunenberg constant

In our opinion, the experiment will show plenty of the mixed L-shapes and T-shapes, with quite rare inclusions of the spiral and planar shapes. This in turn presents a possibility of a self-assembly of a layered structure upon the self-assembled non-planar structures. This hypothesis will be tested in our future study.

For the first time, from the first principles, we show that the conformational capacity of the nucleic base pairs is much richer from the perspective of the self-assembly than it is used to consider in the modern nanobiotechnology. The capability of the modified cytosine and the modified thymine to create the significantly non-planar structures opens the way for the novel “stereo approach” to construction of the nanobiotechnological devices. We believe that the modern nanobiotechnological basis can and should be enriched by the new nucleic base pairs with an innate ability for the non-planar structures.

We think there might be at least three possible ways of a future development of the “stereo approach”.

The first way is to find the other non-planar nucleobase pairs that would be much more convenient for usage in a real industry than those that we have found and presented in this paper. The next way might be searching for an appropriate nucleobase modification in order to achieve the same goal. And the third way is to try to synthesize similar to the non-planar nucleobase pair structures which would behave themselves both as non-planar and energetically favorable at the same time.

## Abbreviations

AIM: atoms in molecules
BCP: bond critical point
DFT: density functional theory
DNA: deoxyribonucleic acid
m^1^Cyt: 1-methylcytosine
m^1^Thy: 1-methylthymine
NBO: natural bond orbital
QTAIM: quantum theory: atoms in molecules
RNA: ribonucleic acid
